# Neurogenesis-Associated Protein, a Potential Prognostic Biomarker in Anti-PD-1 Based Kidney Renal Clear Cell Carcinoma Patient Therapeutics

**DOI:** 10.3390/ph17040451

**Published:** 2024-03-30

**Authors:** Rui Gao, Zixue Liu, Mei Meng, Xuefei Song, Jian He

**Affiliations:** 1 State Key Laboratory of Systems Medicine for Cancer, Center for Single-Cell Omics, School of Public Health, Shanghai Jiao Tong University School of Medicine, Shanghai 200025, China; muzili.rui@sjtu.edu.cn (R.G.); zixuel77@sjtu.edu.cn (Z.L.); mm2020@sjtu.edu.cn (M.M.); 2 Department of Ophthalmology, Ninth People’s Hospital, Shanghai Jiao Tong University School of Medicine, Shanghai 200025, China

**Keywords:** TKTL1, tumor infiltration lymphocytes, prognosis, kidney renal clear cell carcinoma, Biomarker, alkaloids

## Abstract

The transketolase 1 gene (TKTL1) is an essential factor that contributes to brain development. Some studies have shown the influence of TKTL1 in cancers, but it has been rarely reported in kidney cancer. Furthermore, the role of TKTL1 in the prognosis and tumor infiltration of immune cells in various cancers, particularly kidney cancer, remains unknown. In this study, TKTL1 expression and its clinical characteristics were investigated using a variety of databases. TIMER was used to investigate the relationship between TKTL1 and immune infiltrates in various types of cancer. We also studied the relationship between TKTL1 expression and response to PD-1 blocker immunotherapy in renal cancer. We conducted TKTL1 agonists virtual screening from 13,633 natural compounds (L6020), implemented secondary library construction according to the types of top results, and then conducted secondary virtual screening for 367 alkaloids. Finally, in vitro assays of cell viability assays and colony formation assays were performed to demonstrate the pharmacological potency of the screening of TKTL1 agonists. Using these methods, we determined that TKTL1 significantly affects the prognostic potential in different types of kidney cancer patients. The underlying mechanism might be that the TKTL1 expression level was positively associated with devious immunocytes in kidney renal clear cell carcinoma (KIRC) rather than in kidney renal papillary cell carcinoma (KIRP) and kidney chromophobe (KICH). This recruitment may result from the up-regulation of the mTOR signaling pathway affecting T cell metabolism. We also found that TKTL1 may act as an immunomodulator in KIRC patients’ response to anti-PD-1 therapy. Moreover, we also found that piperine and glibenclamide are potent agonists of TKTL1. We have demonstrated, in vitro, that piperine and glibenclamide can inhibit the proliferation and clone formation of Caki-2 cell lines by agonizing the expression of TKTL1. In summary, our discovery implies that TKTL1 may be a promising prognostic biomarker for KIRC patients who respond to anti-PD-1 therapy. Piperine and glibenclamide may be effective therapeutic TKTL1 agonists, providing a theoretical basis for the clinical treatment of kidney cancer.

## 1. Introduction

Kidney cancer is one of the most common cancers in humans. It has been increasing in incidence over the past 20 years, accounting for 2 to 3% of all new tumor cases [1]. The human renal cell carcinoma (RCC) types originate from renal epithelial cells and account for more than 90% of renal cancers [2]. Renal cancer may occur in many different and specific types, possessing attributes that may have different histology, different clinical courses, and different responses to many different treatments. To date, KIRC, KIRP, and KICH are the most common renal cancers [3,4]. Approximately 70% of RCC is localized or locally advanced at diagnosis [5]. Localized RCC can be completely resected by surgery, but previous studies have shown that it usually relapses and has the chance to develop distant metastasis [6]. Patients with metastatic RCC do not respond well to chemotherapy or radiotherapy. Although the introduction of targeted therapy has improved the prognosis of these patients, 5-year acquired resistance is only 10% due to adverse reactions and intrinsic or survival rate [7]. Therefore, it is necessary to identify the genes involved in RCC invasion and metastasis and to clarify their functions. The discovery of antibodies that target immune checkpoints, such as PD-1 and PD-L1, has revolutionized the treatment of RCC over the last decade [8]. Santoni et al. evaluated the treatment outcomes of cancer patients, including non-small cell lung cancer, urothelial cancer, renal cell carcinoma and melanoma, receiving immune checkpoint inhibitors (ICIs) alone or in combination with other agents versus control and found that the use of ICIs may significantly increase the chances of disappearance of all target lesions in response to therapy versus control treatments [9,10,11,12]. Additionally, previous studies have revealed that tumor-infiltrating lymphocytes (TILs) play an important role in modulating chemotherapy response and improving clinical prognosis in various types of cancer [13,14,15], such as tumor-infiltrating neutrophils (TINs) and tumor-associated macrophages (TAMs) [16,17,18], and they also associate with the prognosis [19,20,21,22]. As a result, it is a critical and urgent requirement to understand the immunophenotypes of tumor-immune interactions and identify new immune therapy targets for renal cancers.

Transketolase (TKT) is a rate-limiting enzyme involved in the non-oxidized part of PPP. TKTL1, located on chromosome Xq28, is a regressive enzyme of the non-oxidized part of PPP that generates approximately 60–70% of TKT activity in human colon cancer and liver cells [23]. The non-oxidative pentose phosphate pathway (PPP) is a critical component of cell metabolism because it not only connects glycolytic intermediates and ribo-5-phosphate (R5P) but also regulates the tricarboxylic acid cycle (TCA cycle) of glucose-derived carbon into metabolic tissues [24,25,26]; the TCA cycle has been shown to impair CD8^+^ T by affecting T cell energy metabolism [23,27]. Therefore, we think that TKTL1 affects the energy metabolism of T cells.

Mammalian target of rapamycin (mTOR) is an important signaling pathway of cellular energy metabolism. This pathway can integrate energy, nutrition, and various growth factor signals from inside and outside the cell, participate in the regulation of cell proliferation, metabolism, and survival, etc., is the central regulator of cell growth, and can induce T cell function and differentiation. Some studies have reported that silencing TKTL1 can reduce sphingolipid levels and activate the PI3K/Akt/mTOR signaling pathway [28]. mTORC1 is one of the complexes of mTOR and has an essential function in the mTOR signaling pathway. The correlation between key factors of the mTORC1 metabolic pathway and the TKTL1 gene can further verify the relationship between the TKTL1 gene and T cell energy metabolism.

Natural products have been attractive alternatives for the prevention and treatment of disease throughout human history and have contributed to the development of modern medicines. However, the underlying mechanisms of natural product therapy for tumors remain unclear, especially for kidney cancer. Glibenclamide, a second-generation agent of sulfonylureas, can bind to the sulfonylurea receptor 1 (SUR1), a KATP channel subunit expressed on the plasma membrane of pancreatic beta cells [29].

Piperine is isolated from several members of the piperaceae family, including piper longum, piper nigrum, piper chaba, piper sarmentosum, and piper guineense. Piperine has been identified as a potential anticancer agent that regulates autophagy. In this study, we examined the impact of piperine, glibenclamide, and scopolamine on the growth of cells and the formation of colonies in the Caki-2 cell line and found that piperine and glibenclamide can effectively agonize the activity of TKTL1, indicating that small molecule drugs of kinase agonists may play a role in tumor treatment, achieving drug repositioning and providing novel ideas for kidney cancer treatment.

## 2. Results

### 2.1. TKTL1 mRNA Expression and the Prognosis Analysis

**Figure 1:**
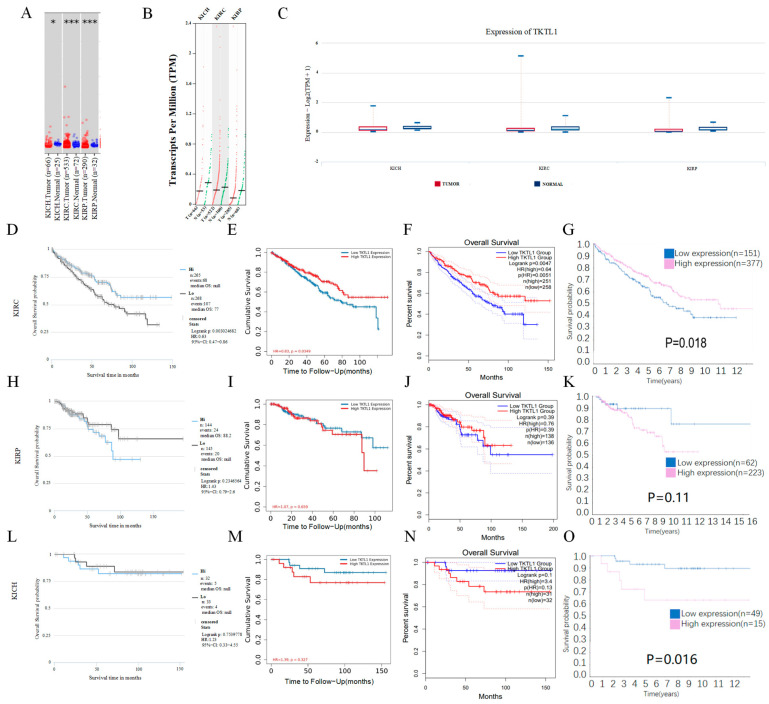
TKTL1 mRNA expression levels in the TIMER (**A**), GEPIA2 (**B**), TCIA (**C**), and the prognosis analysis of TKTL1 in kidney cancer in the Cancer Immunome Atlas (**D**,**H**,**L**), TIMER (**E**,**I**,**M**), GEPIA (**F**,**J**,**N**) and Human Protein Atlas (**G**,**K**,**O**). (**A**) Various TKTL1 expressions in data sets of different cancers via TIMER. Distributions of gene expression levels are displayed using box plots, with the statistical significance of differential expression evaluated using the Wilcoxon test. *p* value: 0 ≤ *** < 0.001, 0.01 ≤ * < 0.05. (**B**) Different TKTL1 mRNA expression patterns analysis via GEPIA2. (**C**) Human TKTL1 expression levels in different cancers from TCGA database via The Cancer Immunome Atlas. Survival curves of OS in kidney renal clear cell carcinoma (**D**–**G**), kidney renal papillary cell carcinoma (**H**–**K**), and kidney chromophobe (**L**–**O**).

The mRNA expression levels of TKTL1 in kidney cancer and adjacent normal tissues were examined by analyzing the TKTL1 mRNA expression levels through the prominent online database TIMER2. In KICH, KIRC, and KIRP, the tumor tissue exhibited a notably reduced expression level of TKTL1 in comparison to the surrounding normal tissues (Figure 1A–C).

We discovered that the manifestation of TKTL1 varied across various types of cancers and exhibited diverse patterns in terms of prognosis, particularly in cases of renal carcinoma (Supplementary Figure S1). Then, we examined the association between TKTL1 expression and outcome in TCIA, TIMER, and GEPIA2 databases. We found that a better prognosis in KIRC was associated with higher TKTL1 expression levels (Figure 1D–F); the groups of individuals with kidney renal clear cell carcinoma showed that a higher level of TKTL1 expression was linked to a more favorable prognosis (OS HR = 0.63, 95% CI = 0.47 to 0.86). However, there was a weak correlation between the level of TKTL1 expression and prognosis in the KIRP and KICH cohorts (Figure 1H–J,L–N, log-rank *p* = 0.2346564; log-rank *p* = 0.7539778). Therefore, we conclude that there is a strong positive correlation between the elevated expression of TKTL1 in KIRC and a favorable prognosis. In contrast, the relationship between KIRP and KICH is not statistically significant. The results of Human Protein Atlas (HPA) showed that a better prognosis is shown to be associated with a higher TKTL1 protein level in KIRC and a lower TKTL1 protein level in KICH, but not in KIRP (Figure 1G,K,O). The data validated the prognostic significance of TKTL1 in certain specific cancer types, wherein the prognostic value of TKTL1 expression varied, depending on the type of cancer in whether it increased or decreased.

### 2.2. TKTL1 Expression Levels Have an Impact on Various Clinical Features of Kidney Cancer

Through examining the correlation between the levels of TKTL1 expression and various clinical characteristics, our goal is to uncover the mechanism and significance of TKTL1 expression levels in different types of cancer, particularly in individuals with varying clinical stages of kidney cancer. We found that the expression level of TKTL1 varied significantly between races and genders of KIRC rather than in KIRP; higher expression of TKTL1 was associated with better OS in female patients and the white race of KIRC (OS HR = 0.5, *p* = 0.0058; OS HR = 0.66, *p* = 0.01). High expression of TKTL1 was also associated with better OS only in the early stage (Stage 1 (OS HR = 0.45, *p* = 0.0072)) rather than in the late stage of the KIRC patient cohort, whereas no such association was found in the KIRP patient cohort (Table 1). The combination of these striking occurrences and the varying survival correlation between KIRC and KIRP shown in Figure 1 suggests a possible link between TKTL1 expression and the prognosis of different cancer types.

### 2.3. Functional Enrichment Analyses of TKTL1

**Figure 2:**
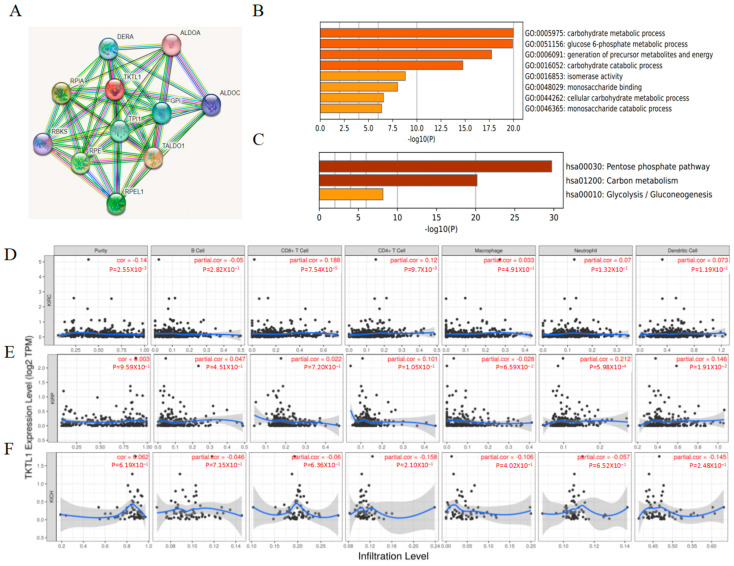
Enrichment analysis of the related genes of TKTL1 and correlation analysis of TKTL1 with immune infiltration in patients with KIRC, KIRP, and KICH. (**A**) Gene–gene interaction network in the STRING database. (**B**) Heatmap of GO enrichment terms colored by *p* values. Orange is the enrichment terms, colored by *p* values. (**C**) Heatmap of Kyoto Encyclopedia of Genes and Genomes (KEGG) enriched terms colored by *p* value. Orange is the enrichment terms, colored by *p* values. (**D**–**F**) Relationship between TKTL1 and immune cells infiltration in KIRC, KIRP, and KICH.

STRING utilizes a spring mechanism to produce the network visuals that encapsulate the network of anticipated connections for a specific set of proteins. The boundaries depict the anticipated functional connections. Fusion evidence is represented by the red line, while neighborhood evidence is represented by the green line, co-occurrence evidence is represented by the blue line, experimental evidence is represented by the purple line, text mining evidence is represented by the yellow line, database evidence is represented by the light blue line, and co-expression evidence is represented by the black line. In this String database, we obtained the 10 proteins closest to TKTL1 from the predictive association network of TKTL1: ALDOA, ALDOC, TALDO1, RPEL1, RPE, RBKS, RPIA, DERA, TPI1, and GPI (Figure 2A).

These interactive genes of TKTL1 were then all input into the Metascape for further gene analysis. We found that the carbohydrate metabolic process, metabolism of glucose 6-phosphate, production of precursor metabolites and energy, carbohydrate catabolic process, isomerase activity, monosaccharide binding, cellular carbohydrate metabolic process, and monosaccharide catabolic process were involved in the regulation of TKTL1 interactive genes (Figure 2B). The analysis of the KEGG pathways revealed a notable enrichment in the pentose phosphate pathway, carbon metabolism, and glycolysis/gluconeogenesis (Figure 2C). These findings showed that TKTL1 serves an essential role in glycometabolism and several other important metabolic processes.

### 2.4. The Immune Infiltration Level in Renal Cancers Is Associated with the Expression Level of TKTL1

It has been demonstrated that TILs can serve as a standalone prognostic factor for cancer survival. In this study, we investigated whether the mRNA expression of TKTL1 is associated with the levels of immune infiltration in different forms of kidney cancer using the TIMER database. We examined the associations between TKTL1 expression and levels of immune infiltration in almost forty cancer types. The findings indicate that the TKTL1 expression level exhibits notable inverse associations with tumor purity across 17 cancer types, which suggests that TKTL1 is connected to the recruitment of lymphocytes to tumors and shows strong positive associations with the levels of B cell infiltration in 15 different cancer types. Furthermore, the expression level of TKTL1 exhibits noteworthy positive associations with the levels of infiltrating CD8+ T cells in 12 different cancer types, 17 types of cancer that involve CD4+ T cells, 14 types of cancer that involve macrophages, 16 types of cancer that involve neutrophils, and 18 types of cancer that involve dendritic cells (Supplementary Table S1 and Figure S2).

After observing the correlation between the expression level of the TKTL1 and immune infiltration levels in various forms of cancer, our subsequent focus was to examine the specific types of cancers where TKTL1 is associated with prognosis and immune infiltration. The assessment of immune infiltration in clinical tumor samples using genomic methods was significantly impacted by the purity of the tumor. We chose the types of cancer in which the levels of TKTL1 expression exhibit a notable inverse relationship with tumor purity in TIMER, as well as a noteworthy direct relationship with prognosis. The tumor purity was significantly negatively correlated with the TKTL1 expression levels in KIRC, but not in KIRP and KICH (Figure 2D–F). TKTL1 mRNA expression level had significant positive correlations with the infiltrating levels of CD4+ T cells, CD8+ T cells in KIRC, neutrophils, and DCs in KIRP (Figure 2D,E). What interested us is that the correlation with immune cells demonstrated a different pattern in KIRC, KIRP, and KICH of kidney cancers. The results strongly indicated that TKTL1 has a distinct function in the infiltration of the immune system in various forms of kidney cancer, resulting in an improved prognosis for KIRC. We further explored the prognostic impact of TKTL1 expression and immune infiltration in KIRC, KIRP, and KICH, and we studied the relationship between different immune cells and kidney cancer OS. We found that TKTL1 and CD8+ T cells, CD4+ T cells, T cell CD4+ memory resting and dendritic cells are positively correlated in KIRC, but not in KIRP and KICH (Supplementary Figure S3), which suggested that the underlying mechanism of TKTL1 leading to a different prognosis pattern in three kidney cancer subtypes might be the recruitment and infiltration of TILs.

### 2.5. TKTL1 Is Involved in T Cell Energy Metabolism in KIRC and KIRP

**Figure 3:**
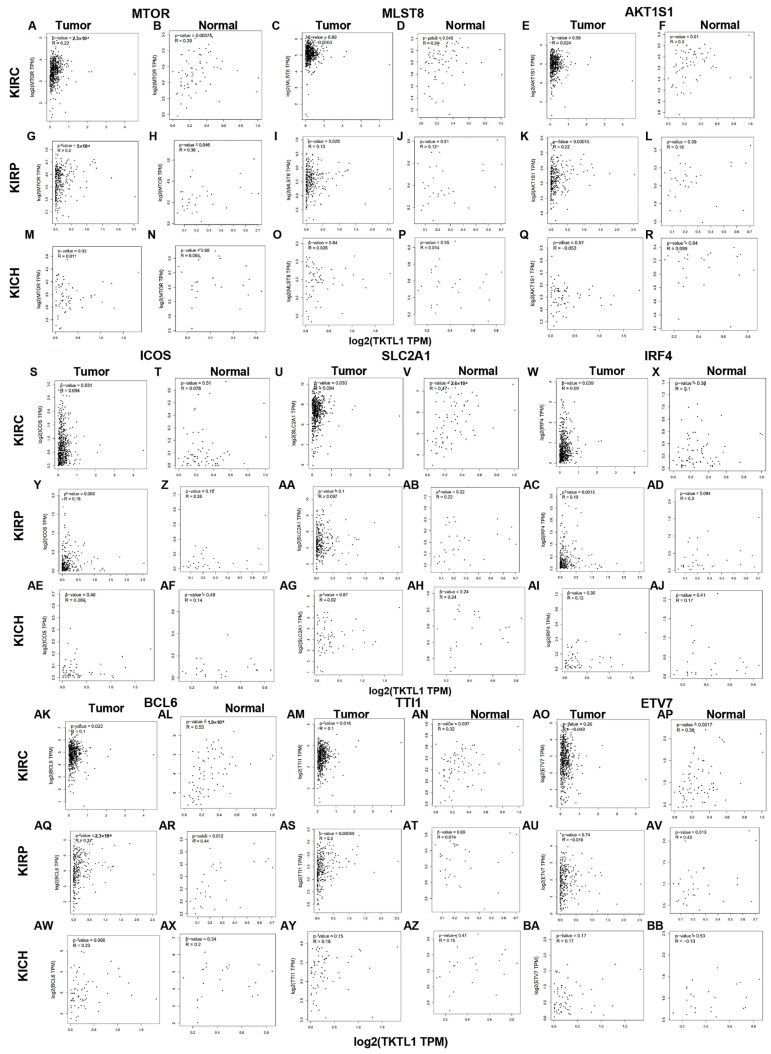
Correlation analysis between TKTL1 expression and genes involved in T cell energy metabolism of renal cancers. Scatterplots of correlations between TKTL1 and MTOR (**A**,**B**), MLST8 (**C**,**D**), AKT1S1 (**E**,**F**), ICOS (**S**,**T**), SLC2A1 (**U**,**V**), IRF4 (**W**,**X**), BCL6 (**AK**,**AL**), Tti1 (**AM**,**AN**), and Tel2 (**AO**,**AP**) of KIRC; scatterplots of correlations between TKTL1 and MTOR (**G**,**H**), MLST8 (**I**,**J**), AKT1S1 (**K**,**L**), ICOS (**Y**,**Z**), SLC2A1 (**AA**,**AB**), IRF4 (**AC**,**AD**), BCL6 (**AQ**, **AR**), Tti1 (**AS**,**AT**), and Tel2 (**AU**,**AV**) of KIRP; scatterplots of correlations between TKTL1 and MTOR (**M**,**N**), MLST8 (**O**,**P**), AKT1S1 (**Q**,**R**), ICOS (**AE**,**AF**), SLC2A1 (**AG**,**AH**), IRF4 (**AI**, **AJ**), BCL6 (**AW**,**AX**), Tti1 (**AY**,**AZ**), and Tel2 (**BA**,**BB**) of KICH.

MTOR, the protein known as the mammalian target of rapamycin, plays a crucial role in regulating cellular energy metabolism. MTOR, MLST8, AKT1S1, ICOS, SLC2A1, IRF4, BCL6, TTI1, ETV7 and other molecules constitute and participate in the mTORC1 metabolic pathway. In tumor tissues of KIRC and KIRP patients, the expression levels of these molecules show a strong positive correlation with the expression of TKTL1, whereas there is hardly any significant correlation in KICH (Figure 3).

### 2.6. KIRC Patients Exhibit Distinct Correlation Patterns between Tumor and Normal Tissue

**Figure 4:**
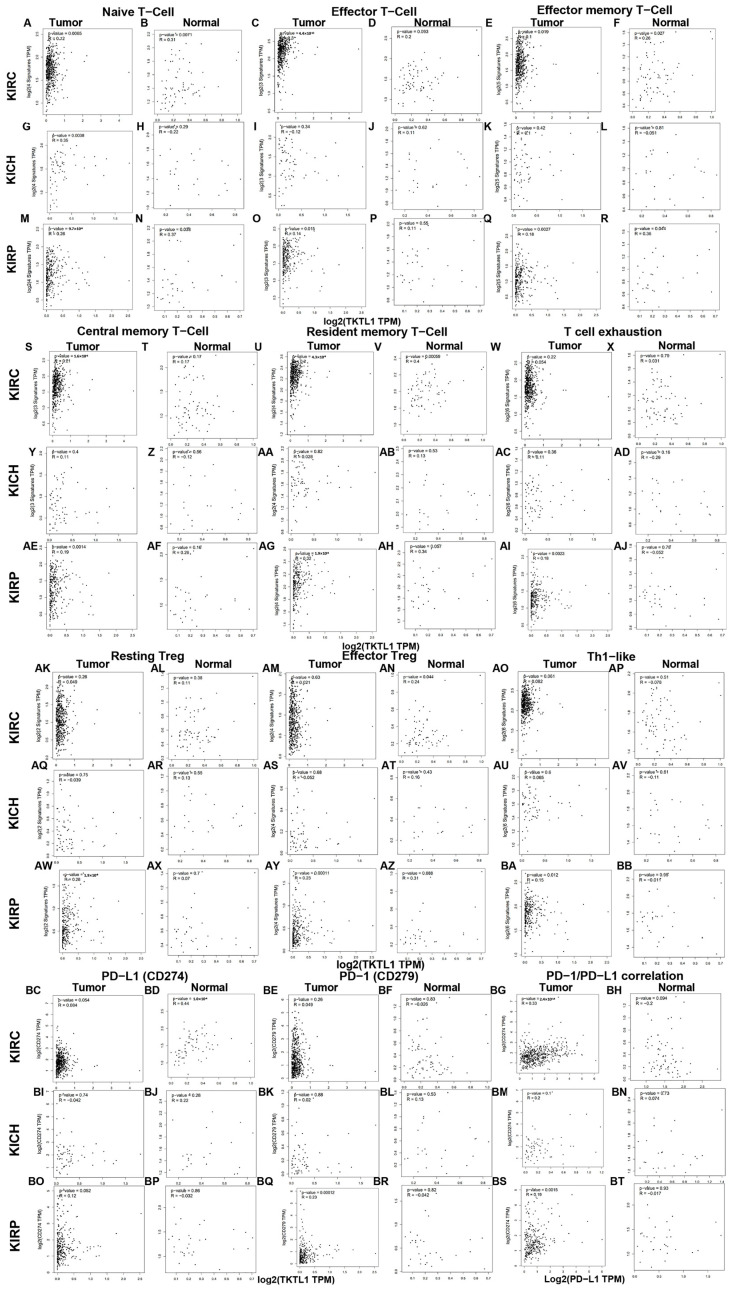
Correlation analysis between TKTL1 and different immune cells and PD-1/PD-L1 in renal cancers. Scatterplots of correlations between TKTL1 and gene markers of naive T cells ((**A**,**B**) of KIRC, (**G**,**H**) of KICH, (**M**,**N**) of KIRP), effector T cell ((**C**,**D**) of KIRC, (**I**,**J**) of KICH, (**O**,**P**) of KIRP), effector memory T cell ((**E**,**F**) of KIRC, (**K**,**L**) of KICH, (**Q**,**R**) of KIRP), central memory T cell ((**S**,**T**) of KIRC, (**Y**,**Z**) of KICH, (**AE**,**AF**) of KIRP), resident memory T cell ((**U**,**V**) of KIRC, (**AA**,**AB**) of KICH, (**AG**,**AH**) of KIRP), T cell exhaustion ((**W**,**X**) of KIRC, (**AC**,**AD**) of KICH, (**AI**,**AJ**) of KIRP), resting Treg ((**AK**,**AL**) of KIRC, (**AQ**,**AR**) of KICH, (**AW**,**AX**) of KIRP), effector Treg ((**AM**,**AN**) of KIRC, (**AS**,**AT**) of KICH, (**AY**,**AZ**) of KIRP), Th1-like ((**AO**,**AP**) of KIRC, (**AU**,**AV**) of KICH, (**BA**,**BB**) of KIRP) cells, and PD-1/PD-L1 axis ((**BC**–**BH**) of KIRC, (**BI**–**BN**) of KICH, and (**BO**–**BT**) of KIRP). Naive T cell: CCR7, LEF1, TCF7, and SELL; effector T cell: CX3CR1, FGFBP2, FCGR3A; effector memory cell: PDCD1, DUSP4, GZMK, GZMA, and IFNG; central memory T cell: CCR7, SELL, and IL7R; resident memory T cell: CD69, ITGAE, CXCR6, and MYADM; T cell exhaustion: HAVCR2, TIGIT LAG3, PDCD1, CXCL13, and LAYN; resting Treg: FOXP3 and IL2RA; effector Treg: FOXP3, CTLA4, and CCR8 TNFRSF9; Th1-like: CXCL13, HAVCR2, IFNG, CXCR3, BHLHE40, and CD4.

It is particularly notable that the majority of marker sets in these immunocytes show significant associations with TKTL1 expression in the tumor tissue of KIRC and KIRP patients. In the KICH, there was no significant correlation between TKTL1 and markers of immune cells (Figure 4). PD-L1/PD1 is an important coinhibitory ligand that inhibits the tumor-killing function of CD8+ T cells, and it is an important mechanism of immune escape of tumor cells. In this study, we found that the correlations between PD-L1/PD1 and TKTL1 expression in three types of kidney cancers (KIRC, KIRP, and KICH) are the same as described above (Figure 4). Regulatory T cells (Treg) are an immunosuppressive subpopulation of CD4+ T cells, which play an important role in maintaining self-tolerance and immune homeostasis. Tregs hinder the immune monitoring of healthy individuals in tumor immunity, inhibiting the host’s antitumor immune response and causing the advancement of tumors in different cancer forms. Hence, Tregs are regarded as a crucial focus for cancer immunotherapy treatment [30]. In the tumor tissues of KIRP patients, we discovered a robust connection between the expression level of a marker set for Resting Treg and TKTL1 expression. However, no notable correlation was observed in KIRC.

The discovery implies that kidney cancer patients exhibit diverse correlation patterns between tumor and normal tissue. The discovery is thrilling as it suggests that TKTL1 could potentially control different kinds of immune cells within the tumor microenvironment of KIRC, making TKTL1 a promising candidate for KIRC treatment.

### 2.7. Predicting the Correlation between TKTL1 Expression and the Effectiveness of PD-1 Blockade Immunotherapy in Individuals Diagnosed with Kidney Cancer

**Figure 5:**
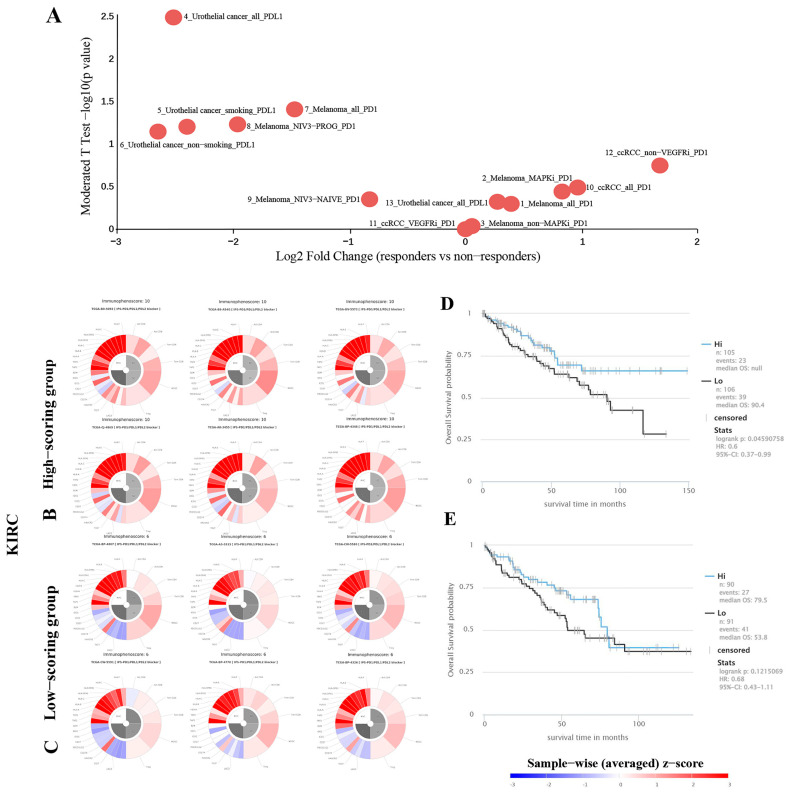
Visualization of differences in TKTL1 gene expression between responders and non-responders to PD-L1 immunotherapy and immunophenoscores, and overall survival analysis of kidney cancer patient’s response to PD-1 blockers immunotherapy in TCIA. (**A**) TKTL1 gene expression exhibited a significant disparity between PD-L1 immunotherapy responders and non-responders. (**B**) Immunophenograms for individual patients with IPS (Immune cell proportion score) >8 of PD-L1 in KIRC. The wheel displays a classification of individual factors into four different categories (clockwise direction): effector cells (EC), suppressor cells (SC), immunomodulators (CP), and MHC molecules (MHC). Effector cells include activated CD4+ T cells, activated CD8+ T cells, effector memory CD4+ T cells, and effector memory CD8+ T cells. Immunosuppressive cells, including MDSC and Treg. The data is displayed by the weighted average z-scores for the elements within the particular category (z > 0; shown in red, z < 0; shown in blue). (**C**) Immunophenograms for patients with IPS < 7 of PD-L1 in KIRC. (**D**) Overall survival of the patients with IPS > 8 in KIRC. (**E**) Overall survival of the patients with IPS < 7 in KIRC.

Analyzing the transcriptome and genome of tumor biopsies that were pretreated to distinguish between individuals who responded positively and those who did not to immunotherapy. The information was utilized to discover patterns and processes of reaction to checkpoint blockade (e.g., anti-PD-L1 and anti-PD-1). We discovered a notable disparity in the expression of the TKTL1 gene between individuals who responded positively to PD-L1 immunotherapy and those who did not (Figure 5A, Supplementary Table S2).

Because of inherent immune resistance, only a small portion of individuals with cancer experience advantages from anti-PD-1 treatment. The influence of TKTL1 on the response of KIRC cancer patients to anti-PD-1 immunotherapy was investigated using the TCIA database. From the TCIA database, we acquired and evaluated the IPS, which can forecast the effectiveness of PD-1 inhibitors in individuals diagnosed with KIRC (Figure 5). Patients with IPS > 8 and < 7 of PD-L1 were selected for follow-up analysis. Immunophenograms of selected individuals were acquired from IPS > 8 and < 7 in PD-L1-treated patients belonging to the KIRC cohorts (Figure 5). In high-scoring patients with KIRC, we discovered a significant positive correlation between the prognosis and the mRNA expression of TKTL1; However, this association was not observed in low-score patients (Figure 5D,E). The findings once again demonstrate the association between TKTL1 and PD-1, and increasing the expression of TKTL1 in patients who positively respond to anti-PD-1 treatment will enhance the chances of survival.

### 2.8. TKTL1 Augments the Benefits of PPARA in KIRC

**Figure 6:**
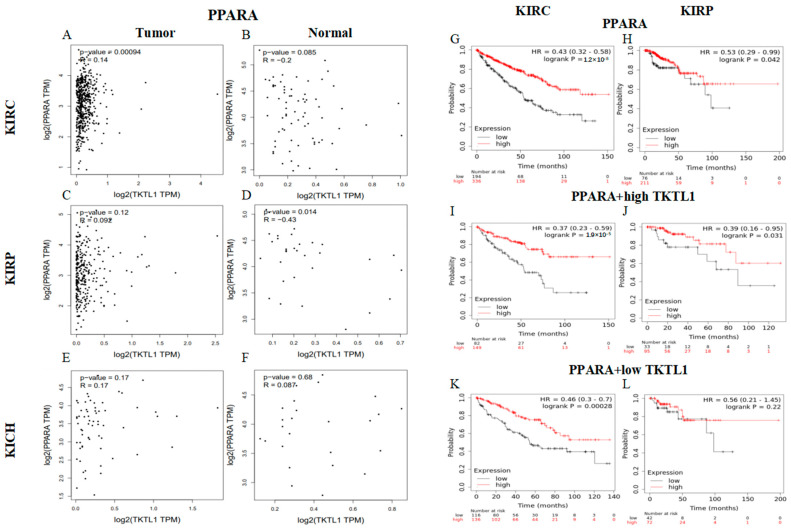
Collaboration between PPARA and TKTL1 contribute to an improved prognosis for KIRC. Scatterplots of correlations between TKTL1 and PPARA of KIRC (**A**,**B**), KIRP (**C**,**D**), and KICH (**E**,**F**). The effect of the expression of PPARA (**G**), PPARA + high TKTL1 (**I**), and PPARA + low TKTL1 (**K**) on overall survival in KIRC. The effect of the expression of PPARA (**H**), PPARA + high TKTL1 (**J**), and PPARA + low TKTL1 (**L**) on overall survival in KIRP.

In the tumor tissue of KIRC patients, we discovered a notable association between PPARA and TKTL1 expression, which is a part of the peroxisome proliferator-activated receptor. However, this correlation was not observed in KIRP and KICH (Figure 6A,C,E). Interestingly, the KIRC cohort showed that PPARA with a high expression of TKTL1 resulted in a better prognosis compared to PPARA alone (OS HR = 0.43; HR = 0.37) (Figure 6G–L). So, there would be better prospects for kidney cancer when PPARA and TKTL1 are co-treated.

### 2.9. Alkaloids Demonstrate Effective Docking Effect with TKTL1

**Figure 7:**
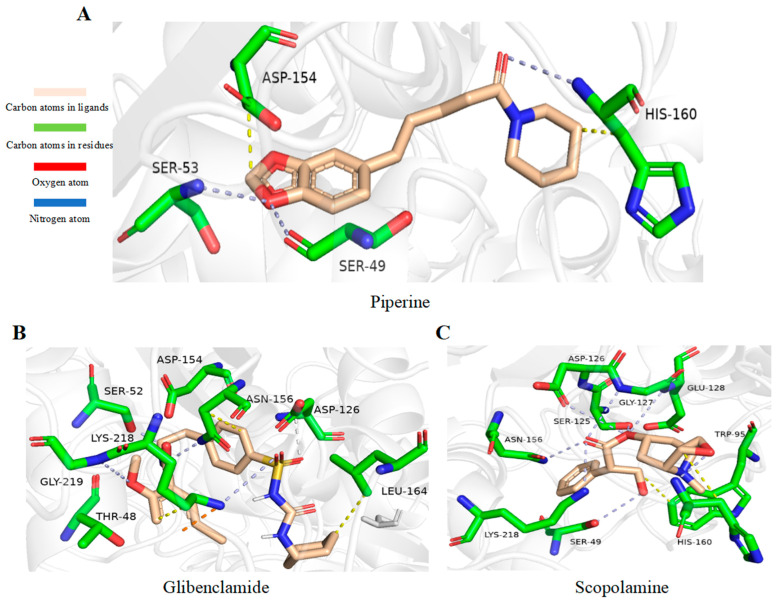
Schematic diagram of molecular docking: (**A**) piperine, (**B**) glibenclamide, and (**C**) scopolamine (Carbon atoms in ligands; shown in brown, Carbon atoms in residues; shown in green, Oxygen atom; shown in red, Nitrogen atom; shown in blue).

Through virtual screening of 13,633 natural product compounds (L6020), we found that alkaloids had a better docking effect with TKTL1. We then built a secondary screening library containing 367 alkaloid compounds and conducted virtual screening again. The results showed that glibenclamide has the energy with the least binding, which means the greatest attraction, indicating that it has the best bonding effect with TKTL1 (Figure 7A–C, Table 2).

By comparing the binding energy, the binding efficiency of glibenclamide, piperine, and scopolamine is comparable, which suggests that these three small molecules have an effective docking effect with TKTL1. At the same time, piperine and scopolamine are both natural product compounds that have the advantages of low drug resistance and high safety.

The results of molecular docking show the patterns of docking action between three small-molecule compounds and TKTL1 (Figure 7A–C) and indicate the names of the residues that form hydrogen bond and hydrophobic bond and their sites of action.

### 2.10. Glibenclamide and Piperine can Effectively Agonize the Activity of TKTL1

**Figure 8:**
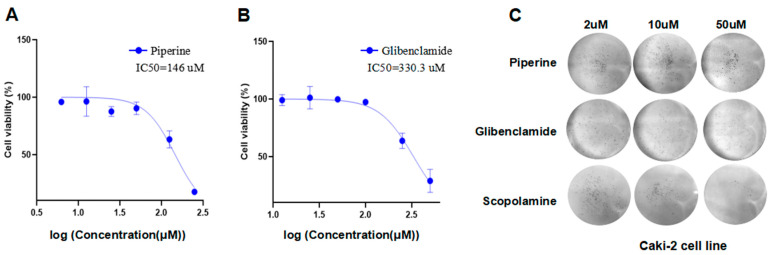
Pharmacological activities of piperine, glibenclamide and scopolamine. (**A**,**B**) Piperine and glibenclamide inhibits the proliferation of Caki-2 cells in vitro through cell counting kit-8 (CCK-8) assay. (**C**) Piperine, glibenclamide, and scopolamine inhibit colony formation of Caki-2 cells.

In this study, we examined the impact of the substances on the growth of cells and the formation of colonies in the Caki-2 cell line. We found that piperine and glibenclamide significantly inhibited cell proliferation with an IC50 value of 146 μM and 330.3 μM (Figure 8A,B). Colony formation assays also showed that piperine, glibenclamide, and scopolamine were effective in inhibiting clone formation in Caki-2 cells at concentrations of 10 μM and 50 μM (Figure 8C).

## 3. Discussion

TKT, along with TKTL1 and TKTL2 constitute the transketolase family that are key enzymes in the non-oxidizing segment of the PPP [31]. However, among these members, only TKTL1 exhibits differential expression in tumor tissues when compared to normal tissues [32,33]. Moreover, TKTL1 has been shown to have connections with various cancers [34,35,36,37]. Despite this knowledge, the precise relationship between TKTL1 and renal cancer, as well as the underlying mechanisms involved, remain inadequately understood.

TKTL1 has been considered a cancer promoter in previous studies and is closely associated with the initiation, development, and progression of cancer. TKTL1 has been found to be associated with promoting lung cancer [34,35], colon cancer and urothelial cancer [33], cervical cancer [36], gastric cancer, and renal cancer [37,38]. In this study, we used various databases for information mining and double-verified that TKTL1 was significantly positively correlated with prognosis in KIRC but not in KIRP and KICH from both gene and protein expression levels. Then, the enrichment analysis of the ten most related TKTL1 proteins found that TKTL1 and its related genes were enriched in the process of glucose metabolism. Through the TIMER database, a robust positive relationship between the expression level of TKTL1 and the level of CD4+ T cells and CD8+ T cells infiltration in KIRC was found. Therefore, considering the correlation between TKTL1 and T cell energy metabolism, we took genes that have been identified to be involved, and we looked at the relationship between TKTL1 expression and KIRC and KIRP in renal cell cancer. We found a positive correlation between KIRC and KIRP, which is exactly the hypothesis that TKTL1 expression is involved in T cell energy metabolism in renal cell cancer. The findings suggest that the immune cell’s infiltration in renal cell carcinoma and adjacent tumors showed a similar pattern. By verification of the GEPIA2 database, we found that patients with KIRC and KIRP had a stronger ability to recruit TILs. However, TKTL1 was significantly positively related to the Treg marker gene in KIRP, but not in KIRC. This also explains the difference between TKTL1 gene expression in KIRC and KIRP on prognosis. Here, we employed independent datasets from TCGA within the TIMER2 platform to ascertain the expression levels of TKTL1 in kidney cancers. We found a significant downregulation of TKTL1 expression in three different kidney cancer types: KICH, KIRC, and KIRP, when compared to normal tissues (Figure 1A–C). In this study, we also found an association between varying levels of TKTL1 and prognostic potential in different cancers. Elevated TKTL1 expression is correlated with improved prognostic outcomes in KIRC (Figure 1D–F). Moreover, higher TKTL1 expression levels were significantly associated with a better prognosis in early-stage kidney cancer (Stage 1 (OS HR = 0.45, *p* = 0.0072)) patients, whereas this association was not evident in the late stage (Table 1). Furthermore, we found that a better OS pattern could only be found in KIRC rather than in KIRP (Table 1). Taken together, these findings strongly indicate that TKTL1 exhibits potential as a prognostic biomarker and protective factor in KIRC. Another crucial aspect highlighted by this study is the observed correlation between TKTL1 and diverse immune enrichment in cancers, especially in KIRC. The enrichment analysis of TKTL1 and related genes showed that they were enriched in the process of glucose metabolism (Figure 2). Since glucose metabolism is an important regulator of T cell function in tumors, TKTL1 was considered to be related to T cell’s immune infiltration. Here, we demonstrate a strong positive association between the level of TKTL1 mRNA expression and CD4+ T cells and CD8+ T cell infiltration in KIRC (Figure 2D). Additionally, it is worth noting that the correlation patterns of immune infiltration levels differ across three types of kidney cancers, namely KIRC, KIRP, and KICH. The correlation observed between TKTL1 and the marker genes of immune cells underscores the potential role of TKTL1 in modulating tumor immunology in these cancers. one possible explanation for this remarkable effect could be that TKTL1 coordinates the function of various immune cells, especially T cell marker gene sets. This evidence supports the assertion that the levels of TKTL1 expression play a significant role in human malignancies and can serve as prognostic indicators for specific cancer types. Firstly, TKTL1 is positively correlated with energy metabolism-related gene expression in T cells. The mTOR signaling pathway is a crucial signaling pathway of cell energy metabolism, promoting substance metabolism and taking part in apoptosis and autophagy, inducing T cell function and differentiation, which plays a significant role in a variety of diseases, such as renal cancer [39]. mTORC1, as a component of mTOR, plays a crucial role in the mTOR signaling pathway. MTOR, MLST8, AKT1S1, ICOS, SLC2A1, IRF4, BCL6, TTI1, ETV7 and other molecules constitute and participate in the mTORC1 metabolic pathway. However, the expression of these molecules is strongly positively related to the expression of TKTL1 in KIRC and KIRP patients, but there is almost no significant correlation in KICH (Figure 3). T cell energy metabolism is pivotal in tumorigenesis and development. For example, effector T cells are important in controlling cancer development. In contrast, Treg cells generally promote immune suppression in the tumor microenvironment, which is often related to tumor advancement. This may be the underlying reason why TKTL1 recruits T cells in KIRC and has different prognostic relevance in the three types of renal cancer. Secondly, in KIRC, KIRP, and KICH, TKTL1 expression levels demonstrated distinct correlations with the regulation of diverse immune cells. For example, the effector T cell is strongly related to TKTL1 in KIRC and KIRP, whereas no significant associations are observed in KICH (Figure 4). Treg inhibits the host antitumor immune response by impairing the immune surveillance of healthy individuals against cancer, leading to tumor progression of various types of cancer. Therefore, Tregs are considered important therapeutic targets for tumor immunotherapy [30]. Here, we found a strong relationship between the resting Treg marker set and TKTL1 in tumor tissues from KIRP patients, whereas no such association was detected in KIRC, which further confirmed that high expression of TKTL1 resulted in a better prognosis of KIRC patients, but not in KIRP. 

Thirdly, in the two renal cell carcinoma subtypes, KIRC and KIRP, the expression of TKTL1 and PD-L1 showed different correlation patterns. PD-L1 is an important coinhibitory ligand that inhibits the tumor-killing function of CD8+ T cells, and it is an important mechanism of the immune escape of tumor cells. Because of inherent immune resistance, only a small fraction of cancer patients experience favorable outcomes from anti-PD-1 therapy. We found a positive relationship between PD-L1 and TKTL1 in KIRC but not in KIRP. Then, we evaluated the prognostic significance of TKTL1 expression in PD-L1-treated patients with immunophenoscores (IPS) >8 and <7 of KIRC, KICH, and KIRP, which were deemed potential responders to PD-1 antibody therapy. A subset of representative patients was chosen to obtain the corresponding immunophenotype map from the TCIA database. We discovered a notable positive association between TKTL1 and prognosis in KIRC patients with high immunophenoscores (Figure 5). All the above findings suggest that TKTL1 may act as an immunomodulator in the anti-PD-1 therapy responder. In addition, PPARA, belonging to the peroxisome proliferator-activated receptor family, promotes cell differentiation, participates in energy metabolism, and suppresses inflammatory responses. We also demonstrate that there would be better prospects for kidney cancer when PPARA and TKTL1 are co-treated (Figure 6). In conclusion, TKTL1 has the potential to be utilized as an independent biomarker for the prognostic evaluation of KIRC patients and can also assist anti-PD-1 therapy in KIRC treatment. Therefore, it is of great significance to screen compounds targeting TKTL1 as combination drugs for anti-PD-1 therapy. To further verify our findings, we obtained TKTL1 agonists, piperine and glibenclamide, from the natural compound library through virtual screening. E-cadherin, a widely recognized suppressor of metastasis [40], facilitates cell–cell adhesion via Ca2+-dependent homophilic interactions, consequently inhibiting cell migration and dissemination. Furthermore, studies have indicated that soluble E-cadherin fragments formed by E-cadherin cleavage promote tumor progression by facilitating tumor invasion and metastasis [41]. According to reports, soluble E-cadherin can promote the invasion, proliferation, and survival of tumor cells, thus playing a pro-cancer role [42]. The transmembrane protein 52B (TMEM52B) is abundantly expressed in normal human tissues and highly expressed in kidneys. A study found that reduced TMEM52B expression was associated with unfavorable survival outcomes and von Hippel-Lindau mutation in patients with renal cancer, suggesting the downregulation of TMEM52B expression and the downregulation of TMEM52B in the occurrence of renal cancer [43]. TMEM52B enhances the stability of E-cadherin by binding to the E-cadherin protein, thereby inhibiting its proteasomal degradation and extracellular shedding.

Up-regulation of E-cadherin leads to increased cell–cell adhesion, which inhibits cell motility and β-catenin activation, thereby inhibiting the development of malignancies. A study found that piperine caused E-cadherin cleavage and decreased β-catenin expression by inhibiting Helicobacter pylori protease, resulting in reduced β-catenin translocation into the nucleus, thereby reducing the risk of tumorigenesis [44]. Piperine may lead to the downregulation of soluble E-cadherin fragments by inhibiting cleavage of E-cadherin protein, inhibiting cell movement and activation of β-catenin, and thus, inhibiting the development of malignant tumors.

Through cell viability assays and cell colony formation experiments, it was found that piperine and glibenclamide can effectively agonize the activity of TKTL1, indicating that small molecule drugs of kinase agonists may play a role in tumor treatment, achieving drug repositioning and providing novel ideas for kidney cancer treatment (Figure 8). Our work implies that TKTL1 may be a promising prognostic biomarker for KIRC patients that respond to anti-PD-1 therapy, providing insight into the possible role of TKTL1 in tumor immunology and its application as a prognostic biomarker and novel therapeutic target for kidney cancer. However, some limitations still exist. On the one hand, RCC is a malignant solid tumor characterized by heterogeneity, so that the discovery of a single biomarker may not be sufficient to guide the clinical treatment of RCC patients. On the other hand, this study was only validated in cellular experiments, and in the future, we also plan to continue the study of the mechanisms. In recent years, the therapeutic pathway in RCC has been greatly improved with the introduction of immunotherapy, and immunotherapy combined with biomarkers predicting the prognosis of patients with renal cell carcinoma is urgently needed in the future to jointly guide clinical treatment.

## 4. Materials and Methods

### 4.1. Different mRNA Expression in Various Cancer Types and the Prognosis Analysis

The mRNA expression level of TKTL1 in different types of cancer was identified using TIMER, GEPIA2, and The Cancer Immunome Atlas (TCIA) databases. Thecorrelation between the TKTL1 expression and the survival of renal cancer was examined by Kaplan-Meier Plotter, TCIA, TIMER, and GEPIA databases using an extensive collection of cancer microarray and RNA-Seq datasets. In addition, we used Human Protein Atlas (HPA) Version 21.0 to identify the correlation between TKTL1 protein levels and survival rate in KIRC, KIRP, and KICH [45].

### 4.2. Enrichment Analysis

Metascape integrates over 40 gene function annotation databases and provides a variety of visualization methods to make gene function analysis easy [46]. Here, we utilized this database to enrich for TKTL1-related genes obtained from STRING, which aims to collect, score, and integrate all publicly available sources of protein–protein interaction (PPI) data [47].

### 4.3. Immune Infiltrates Level and Gene Correlation Analysis

We studied the expression of TKTL1 in different types of malignant tumors and the correlation between TKTL1 expression level and the abundance of immune infiltrating, including CD8+ T cells, CD4+ T cells, macrophages, B cells, DCs, and neutrophils via gene modules using TIMER [48,49]. In addition, T cell energy metabolism is closely related to the occurrence and development of cancer. We used the GEPIA2 database to analyze the correlation between TKTL1 and T cell energy metabolism genes, such as MTOR, MLST8, AKT1S1, ICOS, SLC2A1, IRF4, BCL6, TTI1, and ETV7.

### 4.4. TISIDB Database Analysis

TISIDB database [50] is a network portal for the study of tumor-immune system interactions involving 988 reported immune-related antitumor genes, molecular assays, high-throughput screening techniques, and multi-omics data on carcinogenicity, and a large resource of immunological data from seven public databases. Here, TISIDB provides us with a visual picture of the difference in TKTL1 gene expression between PD-L1 immunotherapy responders and non-responders.

### 4.5. Immunophenotyping of Renal Cancer Patients

The Cancer Immunome Atlas (TCIA) database [51] enables comprehensive analysis of tumor immune and genetic profiles and includes immunogenomics profiles of 20 cancers from TCGA. By ranking the immunophenoscores (IPS) of KIRC, KICH, and KIRP patients likely to respond to PD-1 antibody therapy and selecting representative patients to obtain the corresponding immunophenotype map, we investigated the impact of TKTL1 expression on prognosis in IPS > 8 and < 7 of PD-L1-treated patients.

### 4.6. PPARA Gene Correlation Analysis and Prognosis Analysis

We used the GEPIA2 database to analyze the correlation between PPARA and TKTL1 in KIRC, KIRP, and KICH. The correlation between the TKTL1 expression and the survival of renal cancer was analyzed using Kaplan–Meier Plotter to investigate the relationship between PPARA gene expression level and the prognoses of the patient using an extensive collection of cancer microarray and RNA-Seq datasets.

### 4.7. Virtual Screening Study

To find the best docking molecules for TKTL1, we used Sailvina v1.0 software to obtain and rank the binding energy scores. We used Autodock 4.2.6 software for molecular docking to determine the best conformation. Firstly, the crystal structure of TKTL1 (PDB ID:3MOS) was acquired from the RCSB Protein Data Bank [52] and prepared by eliminating water molecules and unnecessary ligands and adjusting the number of hydrogens. Next, we conducted the first virtual screening among the 13,633 natural compounds (L6020) and obtained the types of compounds with high docking scores. Docked molecules were ranked by the binding affinity score. Then the secondary screening database was established for secondary virtual screening. The compounds with top docking ratings were selected for molecular docking to verify the screening results. We used Autodock 4.2.6 software [53] for global molecular docking. Using grid spacing 0.375 Å and grid coordinates (X, Y, and Z) axes at 60 × 60 × 60, Autogrid was employed to identify the location of the native ligand at protein binding sites. Using Lamarckian Genetic Algorithm (GA) parameters, a total of 10 runs of the GA criterion were performed, and the resulting binding energy was further analyzed [54].

### 4.8. Cell Viability Assay

Piperin (T3002, TOPSCIENCE, Shanghai, China), glibenclamide (B1296, APExBIO, Houston, TX, USA), and scopolamine (C6426, APExBIO, Houston, TX, USA) were purchased. Caki-2 cells in the logarithmic phase were plated in 96-well plates at a density of 5000 cells per well. After 24 h of seeding, cells were processed with serial dilutions of piperine, glibenclamide, and scopolamine and incubated at 37 °C for 72 h. Then 10 μL of CCK-8 (C0037, Beyotime, Shanghai, China) was added to 96-well plates and incubated for 3 h. The optical density (OD) was then recorded at 450 nm using a microplate reader (Synergy H1, Biotek, Winooski, VT, USA). The mean OD450 of control cells exposed to medium without the test compound was set to represent 100% viability. IC50 values were calculated using GraphPad Prism 9.3.0 software.

### 4.9. Colony Formation Assay

Caki-2 cells in the logarithmic phase were plated into the six-well plate at a concentration of 2000 cells per well in the cell culture medium containing 1% P/S, 10% FBS and added with various concentrations of piperine, glibenclamide and scopolamine (2, 10, and 50 μM), then incubated at 37 °C for 10 days. Subsequently, Caki-2 cells were fixed with 4% polyformaldehyde for 15 min at room temperature and stained using 1% crystal violet for 30 min. Cell clones were finally analyzed.

## 5. Conclusions

The present study presents a hypothesis as to why TKTL1 is associated with immune infiltration levels and prognosis in KIRC but not in KICH and KIRP, that is, by recruiting TILs and regulating T cell energy metabolism via TKTL1. These findings shed light on the potential role of TKTL1 in tumor immunology, highlighting its utility as a prognostic biomarker and an emerging therapeutic target for KIRC. Therefore, glibenclamide and piperine may be effective therapeutic TKTL1 agonists, providing a theoretical basis for the clinical treatment of kidney cancer.

## Figures and Tables

**Table 1:** Correlation of TKTL1 mRNA expression levels and clinical prognostic potential in KIRC and KIRP with various clinicopathological factors.

Clinicopathological Characteristics	Overall Survival (*n* = 1925)					
	KIRC (*n* = 530)			KIRP (*n* = 280)		
	N	Hazard Ratio	*p* Value	N	Hazard Ratio	*p* Value
Gender						
Female	186	0.5 (0.3–0.83)	**0.0058**	76	0.23 (0.03–1.76)	0.12
Male	344	0.73 (0.5–1.09)	0.12	211	1.33 (0.65–2.75)	0.44
Race						
White	459	0.66 (0.47–0.91)	**0.01**	175	0.62 (0.32–1.22)	0.16
Asian	8	---	---	6	---	---
Black/African American	56	0.41 (0.12–1.38)	0.14	56	0.41 (0.12–1.38)	0.14
Stage						
1	265	0.45 (0.25–0.82)	**0.0072**	171	1.86 (0.41–8.48)	0.42
2	57	1.93 (0.59–6.28)	0.27	21	1,756,641,030.03 (0-Inf)	0.16
3	123	1.5 (0.86–2.63)	0.15	61	0.46 (0.17–1.28)	0.13
4	82	0.68 (0.41–1.14)	0.14		---	---

Bold values indicate *p* < 0.05.

**Table 2:** Molecular docking studies of the top alkaloids with TKTL1 (PDB ID: 3MOS).

Compound	Structural Formula	Binding Energy (kcal/mol)
**Glibenclamide**	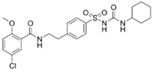	−8.8
**Piperine**	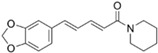	−8.3
**Scopolamine**	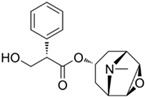	−7.9
**Tropisetron**	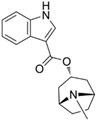	−7.7
**Atropine**	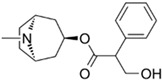	−7.6

## Data Availability

All relevant data are presented in the article and supplementary information files.

## Supplementary Materials

The following supporting information can be downloaded at: https://www.mdpi.com/article/10.3390/ph17040451/s1.
