# Supplementary material for: Neurogenesis-Associated Protein, a Potential Prognostic Biomarker in Anti-PD-1 Based Kidney Renal Clear Cell Carcinoma Patient Therapeutics
Source: Pharmaceuticals (Basel). 2024 Mar 30;17(4):451. doi: 10.3390/ph17040451 (PMC11053496; doi:10.3390/ph17040451)
Supplement: Supplementary file 1 [file pharmaceuticals-17-00451-s001.zip › Supplementary Files/SI_Table1.docx]

Supplementary-Table S1 | The expression level of TKTL1 is correlated with
immune infiltration level in various types of cancers

| Relationship |  | Cancer Type |
| --- | --- | --- |
| Negative  correlations | Tumor purity  (17) | BLCA,BRCA,BRCA-Basal,BRCA-Her2,BRCA-Luminal,CHOL,COAD,ESCA,KIRC,LGG,LUAD,LUSC,MESO,PCPG,PRAD,READ,TGCT |
| Positive  correlations | B cells (15) | BRCA,BRCA-Basal,BRCA-Luminal,ESCA,GBM,HNSC,HNSC-HPVneg,LGG,LIHC,LUAD,LUSC,PAAD,PRAD,TGCT,THCA,UCS |
|  | CD8+ T cells  (12) | BRCA,BRCA-Basal,BRCA-Her2,BRCA-Luminal,HNSC,HNSC-HPVneg,KIRC,LIHC,LUSC,PAAD,TGCT,THCA |
|  | CD4+ T cells  (17) | BRCA,BRCA-Basal,BRCA-Her2,BRCA-Luminal,CESC,COAD,GBM,HNSC,HNSC-HPVneg,KIRC,LGG,LIHC,PAAD,PCPG,PRAD,STAD,THYM |
|  | Macrophages  (14) | BRCA-Basal,CESC,COAD,ESCA,HNSC,HNSC-HPVneg,LGG,LIHC,LUSC,PAAD,PRAD,READ,STAD,THCA |
|  | Neutrophils  (16) | BRCA,BRCA-Her2,BRCA-Luminal,COAD,GBM,KIRP,LGG,LIHC,LUAD,LUSC,PAAD,PCPG,PRAD,READ,SARC,THCA |
|  | Dendritic cells  (18) | ACC,BRCA,BRCA-Luminal,COAD,HNSC,HNSC-HPVneg,KIRP,LGG,LIHC,LUSC,PAAD,PCPG,PRAD,READ,STAD,THCA,THYM,UVM |
