# Supplementary material for: Neurogenesis-Associated Protein, a Potential Prognostic Biomarker in Anti-PD-1 Based Kidney Renal Clear Cell Carcinoma Patient Therapeutics
Source: Pharmaceuticals (Basel). 2024 Mar 30;17(4):451. doi: 10.3390/ph17040451 (PMC11053496; doi:10.3390/ph17040451)
Supplement: Supplementary file 1 [file pharmaceuticals-17-00451-s001.zip › Supplementary Files/SI_Table2.docx]

Supplementary-Table S2 | Expression difference between responders and non-responders

| **No** | **PMID** | **Cancer type** | **Group** | **Drug** | **# Res** | **# NRes** | **Log2 (Fold Change)** | **P value** |
| --- | --- | --- | --- | --- | --- | --- | --- | --- |
| 1 | [26997480](https://www.ncbi.nlm.nih.gov/pubmed/26997480) | Melanoma | all | Anti-PD-1 (pembrolizumab and nivolumab) | 14 | 12 | 0.387 | 0.503 |
| 2 | [26997480](https://www.ncbi.nlm.nih.gov/pubmed/26997480) | Melanoma | MAPKi | Anti-PD-1 (pembrolizumab and nivolumab) | 6 | 5 | 0.833 | 0.356 |
| 3 | [26997480](https://www.ncbi.nlm.nih.gov/pubmed/26997480) | Melanoma | non-MAPKi | Anti-PD-1 (pembrolizumab and nivolumab) | 8 | 7 | 0.044 | 0.957 |
| 4 | [28552987](https://www.ncbi.nlm.nih.gov/pubmed/28552987) | Urothelial cancer | all | Anti-PD-L1 (atezolizumab) | 9 | 16 | -2.512 | 0.00333 |
| 5 | [28552987](https://www.ncbi.nlm.nih.gov/pubmed/28552987) | Urothelial cancer | smoking | Anti-PD-L1 (atezolizumab) | 5 | 9 | -2.4 | 0.0631 |
| 6 | [28552987](https://www.ncbi.nlm.nih.gov/pubmed/28552987) | Urothelial cancer | non-smoking | Anti-PD-L1 (atezolizumab) | 4 | 7 | -2.648 | 0.0723 |
| 7 | [29033130](https://www.ncbi.nlm.nih.gov/pubmed/29033130) | Melanoma | all | Anti-PD-1 (nivolumab) | 26 | 23 | -1.479 | 0.039 |
| 8 | [29033130](https://www.ncbi.nlm.nih.gov/pubmed/29033130) | Melanoma | NIV3-PROG | Anti-PD-1 (nivolumab) | 15 | 11 | -1.962 | 0.0592 |
| 9 | [29033130](https://www.ncbi.nlm.nih.gov/pubmed/29033130) | Melanoma | NIV3-NAIVE | Anti-PD-1 (nivolumab) | 11 | 12 | -0.833 | 0.448 |
| 10 | [29301960](https://www.ncbi.nlm.nih.gov/pubmed/29301960) | Clear cell renal cell carcinoma (ccRCC) | all | Anti-PD-1 (nivolumab) | 4 | 8 | 0.96 | 0.32 |
| 11 | [29301960](https://www.ncbi.nlm.nih.gov/pubmed/29301960) | Clear cell renal cell carcinoma (ccRCC) | VEGFRi | Anti-PD-1 (nivolumab) | 2 | 0 | 0 | 1 |
| 12 | [29301960](https://www.ncbi.nlm.nih.gov/pubmed/29301960) | Clear cell renal cell carcinoma (ccRCC) | non-VEGFRi | Anti-PD-1 (nivolumab) | 2 | 8 | 1.672 | 0.178 |
| 13 | [29443960](https://www.ncbi.nlm.nih.gov/pubmed/29443960) | Urothelial cancer | all | Anti-PD-L1 (atezolizumab) | 68 | 230 | 0.279 | 0.489 |
