# Supplementary figures and images for: Neurogenesis-Associated Protein, a Potential Prognostic Biomarker in Anti-PD-1 Based Kidney Renal Clear Cell Carcinoma Patient Therapeutics
Source: Pharmaceuticals (Basel). 2024 Mar 30;17(4):451. doi: 10.3390/ph17040451 (PMC11053496; doi:10.3390/ph17040451)

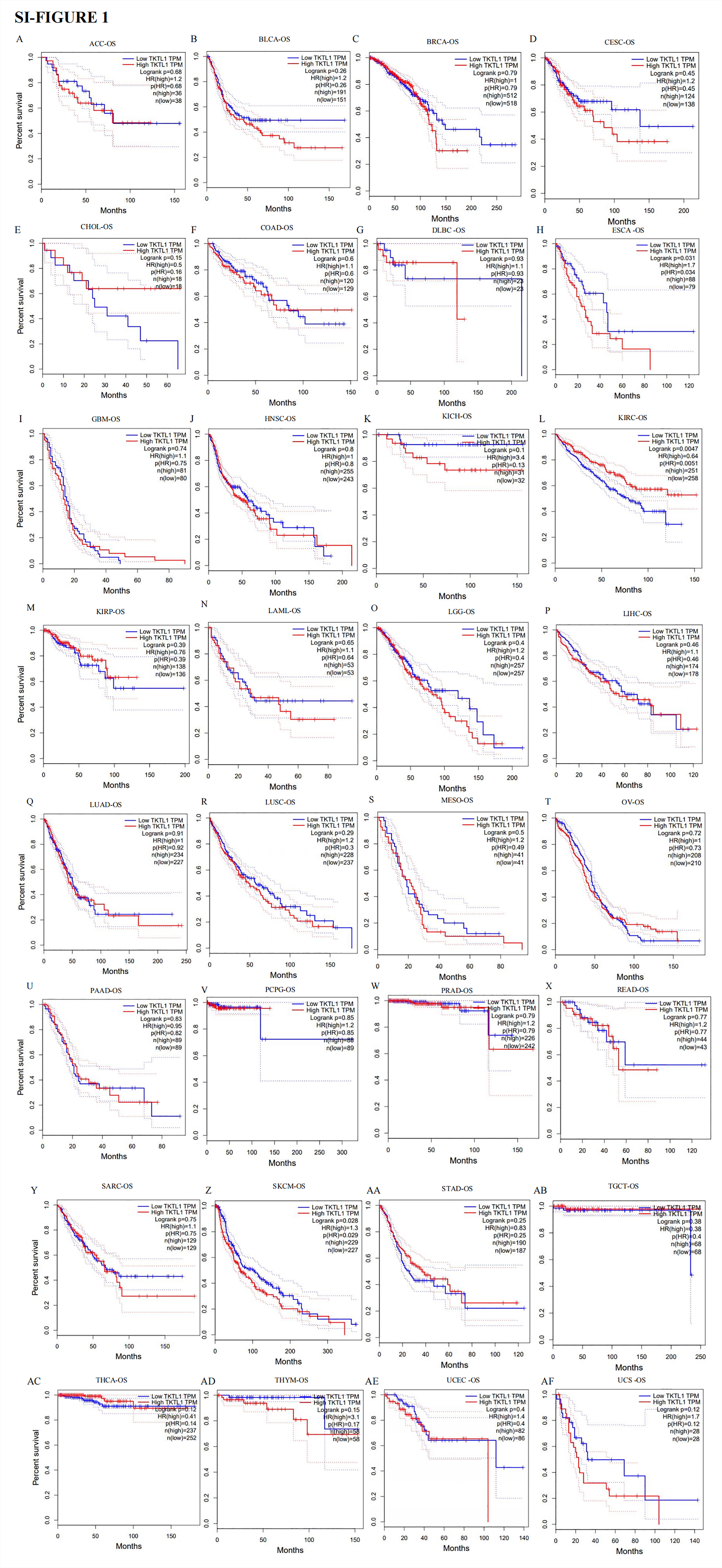

Supplement: Supplementary file 1 [file pharmaceuticals-17-00451-s001.zip › Supplementary Files/SI_Figure 1.tif]

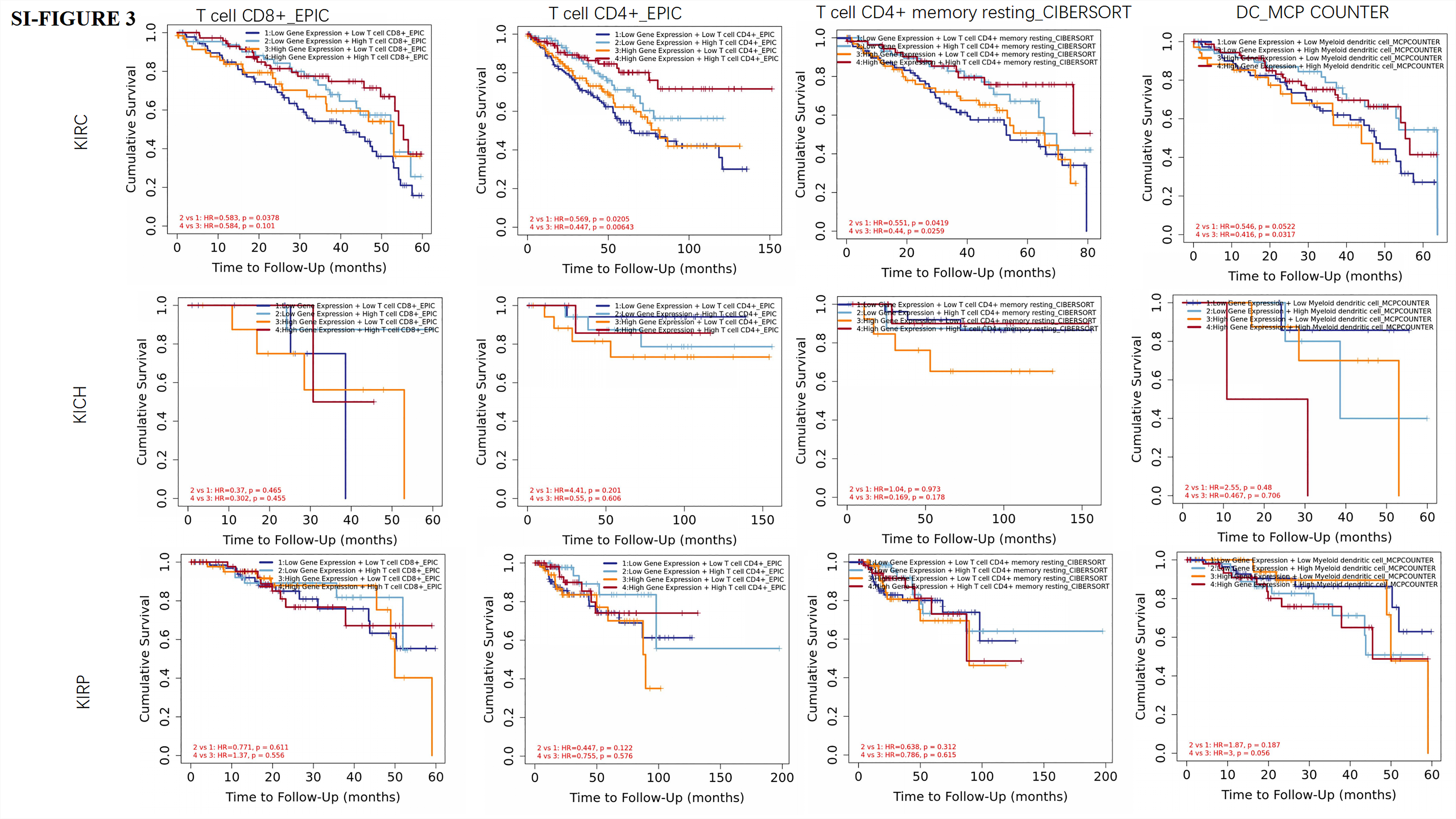

Supplement: Supplementary file 1 [file pharmaceuticals-17-00451-s001.zip › Supplementary Files/SI-Figure 3.tif]
